# Metabolic disturbances in synovial fluid are involved in the onset of synovitis in heifers with acute ruminal acidosis

**DOI:** 10.1038/s41598-019-42007-1

**Published:** 2019-04-01

**Authors:** Pablo Alarcon, Alejandra I. Hidalgo, Carolina Manosalva, Raul Cristi, Stefanie Teuber, Maria A. Hidalgo, Rafael A. Burgos

**Affiliations:** 1 Laboratory of Inflammation Pharmacology, Faculty of Veterinary Science, Institute of Pharmacology and Morphophysiology, Universidad Austral de Chile, Valdivia, Chile; 2 Faculty of Sciences, Institute of Pharmacy, Universidad Austral de Chile, Valdivia, Chile

## Abstract

Acute ruminal acidosis (ARA) is the result of increased intake of highly fermentable carbohydrates, which frequently occurs in dairy cattle and is associated with aseptic polysynovitis. To characterise the metabolic changes in the joints of animals with ARA, we performed an untargeted gas chromatography–mass spectrometry (GC-MS)-based metabolomic analysis of synovial fluid. Seven heifers were challenged with an intraruminal oligofructose overload (13 g/kg of body weight [BW]) dissolved in water. Synovial fluid samples were collected at 0, 9 and 24 h post-overload. Metabolome analysis revealed the presence of 67 metabolites. At 9 h post-overload, glyceric acid, cellobiose, fructose and lactic acid were all increased, whereas at 24 h, sorbitol, lactic acid and fructose levels were all increased >10-fold. At 24 h, citric acid and threonine levels were significantly reduced. We detected increased L- and D-lactate, and the presence of interleukin-6 (IL-6) in synovial fluid. Furthermore, using bovine fibroblast-like synoviocytes, we observed that D-lactate induces IL-6 synthesis. Our results suggest that ARA produces severe metabolomic changes in synovial fluid, including disturbances in starch and sucrose metabolism, and increased lactate levels. These changes were observed prior to the appearance of synovitis, suggesting a potential role in the onset of polysynovitis.

## Introduction

Acute ruminal acidosis (ARA) is a metabolic-nutritional disease that affects cattle^1,2^ and is caused by a high intake of non-structural carbohydrates that are fermented in the rumen by amylolytic bacteria, thereby producing pyruvate and volatile fatty acids (VFA)^3^. The reduction in the pH of ruminal fluid kills gram-negative bacteria, including lactolytic bacteria^3^. This ruminal environment favours an increase in gram-positive lactate-producing bacteria (e.g*., Streptococcus bovis*) and the growth of pH-resistant bacteria such as *Lactobacilli spp*.^4^, which both increase D- and L-lactate concentrations that are absorbed via the ruminal wall into the blood circulation and thereby causes metabolic acidosis^5^. In this regard, a significant increase in D-lactic acid (4.8 mM at 26 h) in the blood has been observed in animals treated with intraruminal glucose at 12 g/kg of body weight (BW), in comparison with a slight increase in L-lactic acid^5^.

This metabolic imbalance is also associated with lameness, a clinical finding frequently related to the incidence of diseases of the locomotor apparatus^6^. Nonpregnant dairy heifers with ruminal acidosis induced by oligofructose overload show tarsocrural joint distension, and in some animals, claw pain^7^. In addition, these animals develop generalised sterile neutrophilic polysynovitis 24 h after oligofructose overload^8^. The origin of this inflammatory condition is yet unknown.

In human joint diseases, high levels of lactate have been found in the synovial fluid of patients with septic arthritis^9,10^, osteoarthritis^11^, osteonecrosis^12^, rheumatoid arthritis (RA) and gout^13,14^. In the joints of patients with RA, fibroblast-like synoviocytes (FLS) produce high concentrations of lactic acid, which has been proposed to be key in the intracellular signalling pathway that controls pro-inflammatory cytokine production^15^, suggesting that metabolic changes are closely associated with joint inflammation.

It has been suggested that changes in the metabolic profile can be useful to discovering new links between inflammation and metabolism. Metabolomics is a new diagnostic approach that has shown promise in identifying and characterising metabolites associated with the inflammatory process^16^, and has the potential to detect 40–150 chemical entities in a single run through gas chromatography–mass spectrometry (GC-MS). Metabolic fingerprints have been characterised in patients with synovitis, RA and osteoarthritis, suggesting that inflammatory diseases can produce a myriad of metabolic changes^17,18^.

The present study shows several metabolomic changes in the joint of a heifer with ARA induced by an overload of oligofructose, resulting in increased levels of several metabolites, including lactate, in synovial fluid. An *in vitro* experiment revealed that D-lactate exerted a direct pro-inflammatory effect on bovine FLS.

## Results

### Intraruminal oligofructose overload induced ARA in heifers

Oligofructose overload (13 g/kg) induced a significant reduction in the pH of ruminal fluid, as observed at 9 and 24 h after challenge (Table 1). The pH values recorded were <5.0, and reduced ruminal contractions and loose stools were commonly observed. These clinical findings agree with those of other authors^19,20^ and diagnosed cases of ARA^21^.

**Table 1:** The pH of ruminal and synovial fluid during oligofructose overload. The arithmetic mean and standard deviation of pH values are shown.

Sample	0 h	9 h	24 h
**Ruminal fuid**	7.1 ± 0.1^a^	4.5 ± 0.1^b^	4.2 ± 0.4^b^
**Synovial fluid**	8.0 ± 0.2^a^	7.7 ± 0.2^a^	7.5 ± 0.1^b^

Different letters indicate significant differences compared at 0 h.

### Synovial fluid from heifers with ARA showed reduced pH and neutrophil recruitment

**Figure 1:**
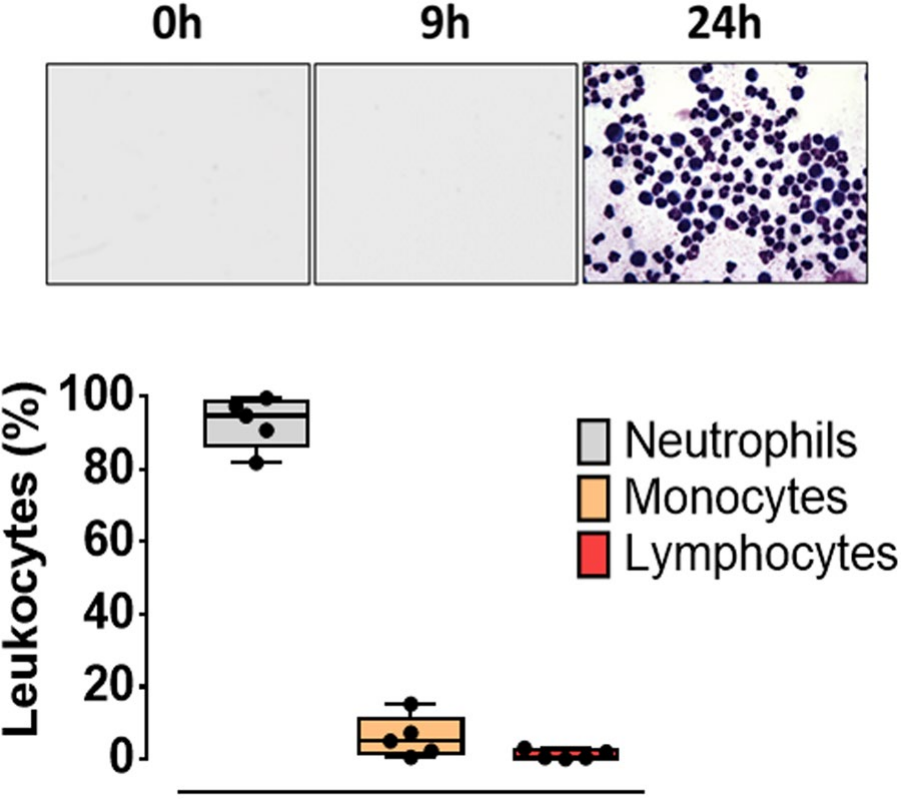
The presence of neutrophils in the joint at 24 h after oligofructose overload. In the upper panel, a representative synovial fluid smear from the tarsocrural joint is shown. A box and whisker plot shows the differential count of leukocytes in the synovial fluid of heifers with acute ruminal acidosis (ARA) at 24 h.

The ARA disorder significantly reduced the pH of synovial fluid 24 h after oligofructose overload (Table 1). The appearance of the synovial fluid was altered only at 24 h, appearing cloudy and viscous and having an abundance of leukocytes (810.4 ± 335.8 cells per field, mean ± S.E.M.). The differential count of synovial fluid smears totalled 92.8% ± 3.1 neutrophils, 6.0% ± 2.6 monocytes and 1.2% ± 0.6 lymphocytes (Fig. 1).

### Metabolome of the synovial fluid of heifers

**Figure 2:**
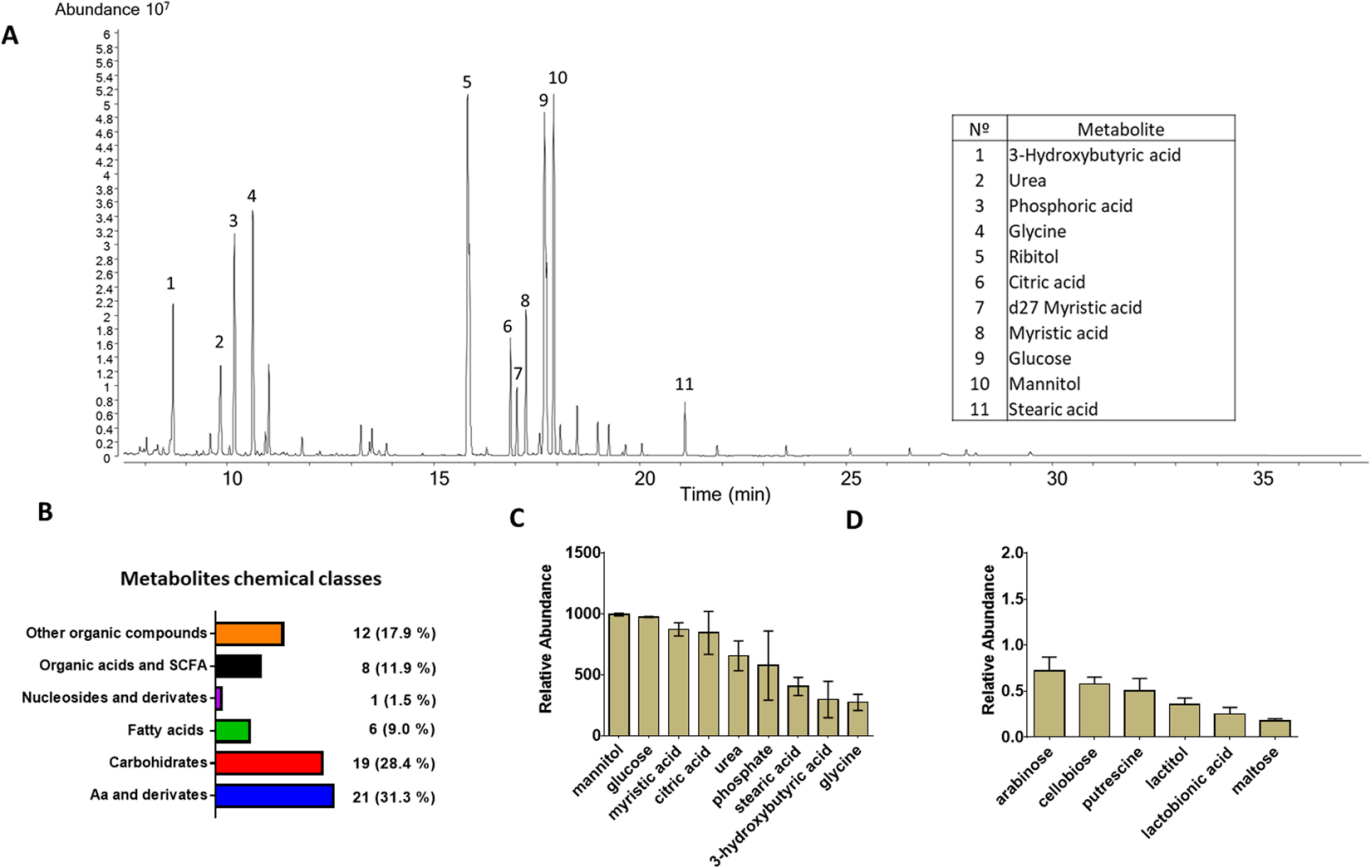
Metabolomic profile of synovial fluid before induction of acute ruminal acidosis (ARA). In (**A**) a representative chromatogram is shown. (**B**) Metabolic entities detected by gas chromatography–mass spectrometry (GC-MS). (**C**) Nine more abundant metabolites in synovial fluid. (**D**) Six less abundant metabolites in synovial fluid. Each bar represents the mean ± S.E.M.

A total of 81 peaks were integrated after GC-MS analysis of synovial fluid, which included internal standards. Among these peaks, 67 metabolites were identified and categorised according to their major chemical classes, including amino acids, peptides/derivatives, carbohydrates, fatty acids, nucleosides/derivatives, organic acids/short fatty acids, and other organic and inorganic compounds (Supplementary Table S1). A characteristic GC-MS chromatogram of synovial fluid and peaks of some metabolites are shown in Fig. 2A. The metabolites identified in the current study were detected using both the retention index according to the fatty acid methyl esters (FAME) standard and the Fiehn library. In addition, retention time, target ion, quantitative ions and chemical structure of the derivative product of each metabolite were used for identification.

Amino acids and their derivatives were the major compounds found in the synovial fluid; they comprised 21 compounds, including 11 amino acids, such as alanine, beta-alanine, glycine, isoleucine, leucine, lysine, methionine, threonine, phenylalanine, tyrosine and valine. In addition, 10 amino acid derivatives were also detected, including allothreonine, ornithine and oxoproline, among others (Fig. 2B).

Nineteen carbohydrates and their metabolites were identified in synovial fluid, including glucose, mannitol, galactose, fructose, lyxose, sucrose, galactitol and arabinose, among others (Fig. 2B). Six fatty acids were detected, including myristic acid, stearic acid, palmitic acid, heptadecanoic acid, pelargonic acid and oleic acid (Fig. 2B). Eight organic acids and short-chain fatty acids (SCFAs), including citric acid, lactic acid, pyruvic acid, glyceric acid, 2-hydroxyglutaric acid, 3-hydroxybutyric acid, 2-hydroxybutanoic acid and 3-hydroxypropionic acid were detected (Fig. 2B). Other organic compounds were also detected, including erythritol, dehydroascorbic acid, hexitol, butane-2,3-diol and propane-1,3-diol (Fig. 2B).

Glucose and mannitol were the most predominant metabolites in synovial fluid. Other predominant metabolites, based on their abundance ratios, were myristic acid, citric acid, urea, phosphate, stearic acid, 3-hydroxybutyric acid and glycine (Fig. 2C). Arabinose, cellobiose, putrescine, lactitol, lactobionic acid and maltose were the six least predominant metabolites identified in the synovial fluid of heifers (Fig. 2D).

### Metabolome changes in synovial fluid after the induction of ARA

**Figure 3:**
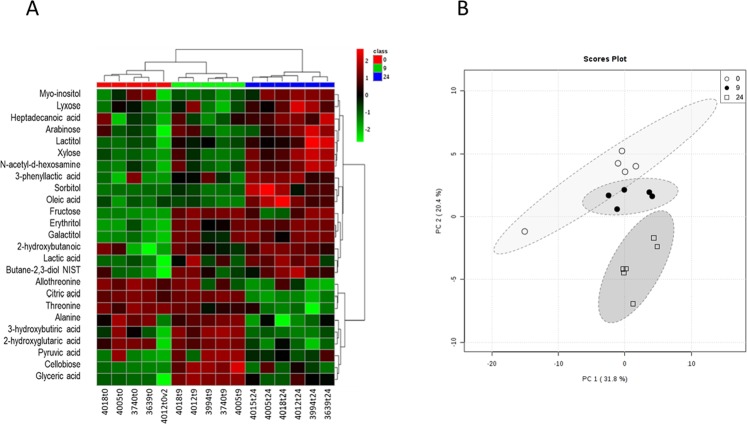
Heatmap and principal component analysis (PCA) of synovial fluid during the induction of acute ruminal acidosis (ARA). In (**A**) a heatmap of 25 metabolites with the lowest p-values, obtained by ANOVA, are depicted. In (**B**) PCA of synovial fluid at 0, 9 and 24 h after the induction of ARA.

To provide intuitive visualisation of normalised metabolome data, a heatmap plot of the 25 compounds with the lowest p-values, as determined by ANOVA, was constructed. A clear hierarchical separation between the different times of ruminal acidosis induction was observed (Fig. 3A). In addition, when an unsupervised multivariate analysis was performed, principal component analysis (PCA) revealed that axes 1 and 2 accounted for 31.8% and 20.4% of the total variation, respectively (Fig. 3B). In the supervised multivariate analysis, partial least squares-discriminant analysis (PLS-DA) showed a distinctive separation between different time points after the induction of ruminal acidosis, as axes 1 and 2 accounted for 23.8% and 18.7% of the total variation, respectively (Supplementary Fig. S1A). In this case, six metabolites (*i.e*., cellobiose, glyceric acid, fructose, erythritol, galactitol and pyruvic acid) with the greatest variable importance in projection (VIP) scores (>1.8) contributed most significantly to the observed separation, as potential biomarkers of synovitis induced by ARA (Supplementary Fig. S1B).

**Figure 4:**
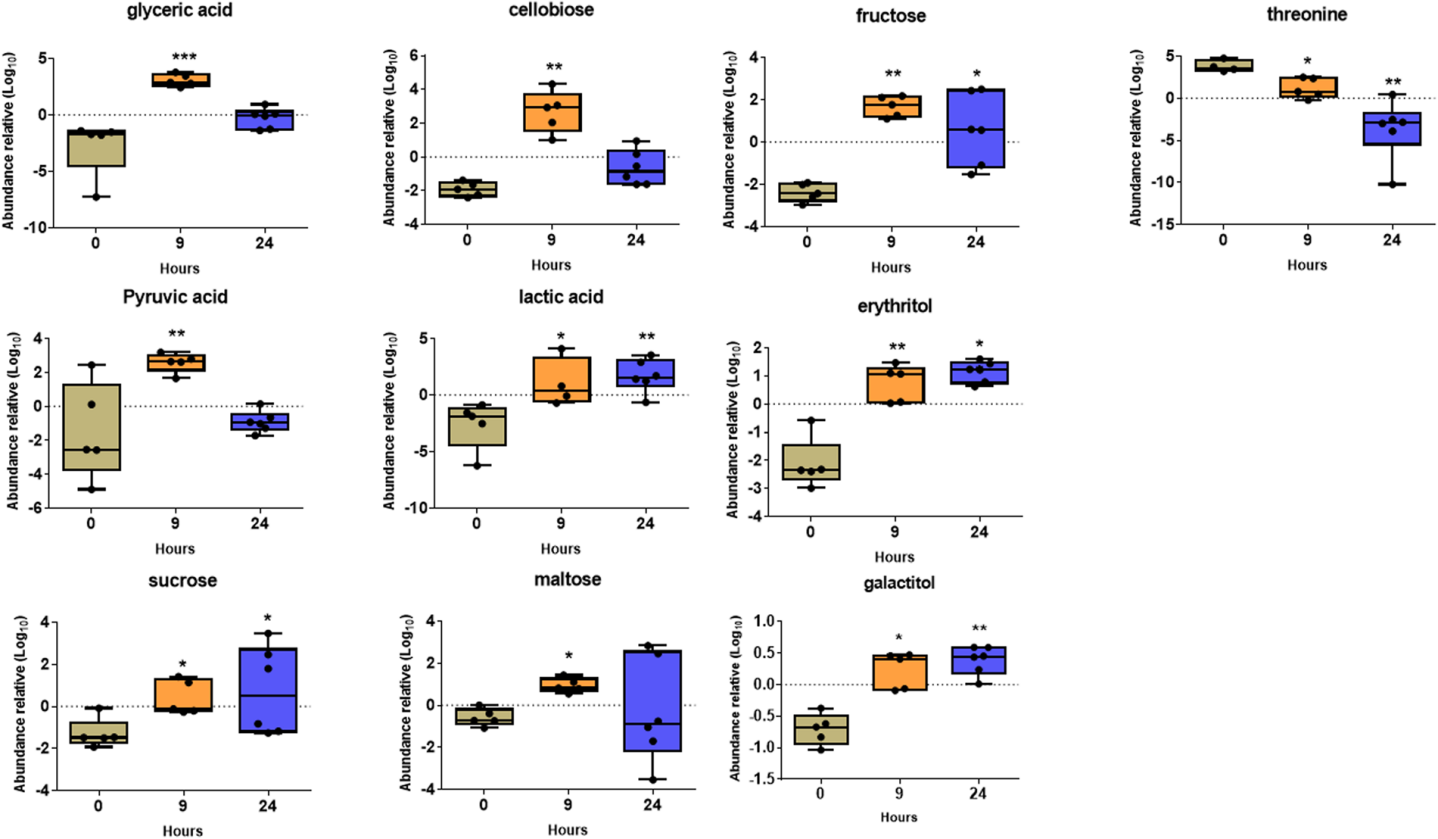
Changes in the relative abundance of metabolites in synovial fluid 9 h after the induction of acute ruminal acidosis (ARA). Box and whisker plot showing the median relative abundance. Each point represents an experimental animal. *p < 0.05; **p < 0.01 compared to 0 h; Dunn’s multiple comparison test.

**Figure 5:**
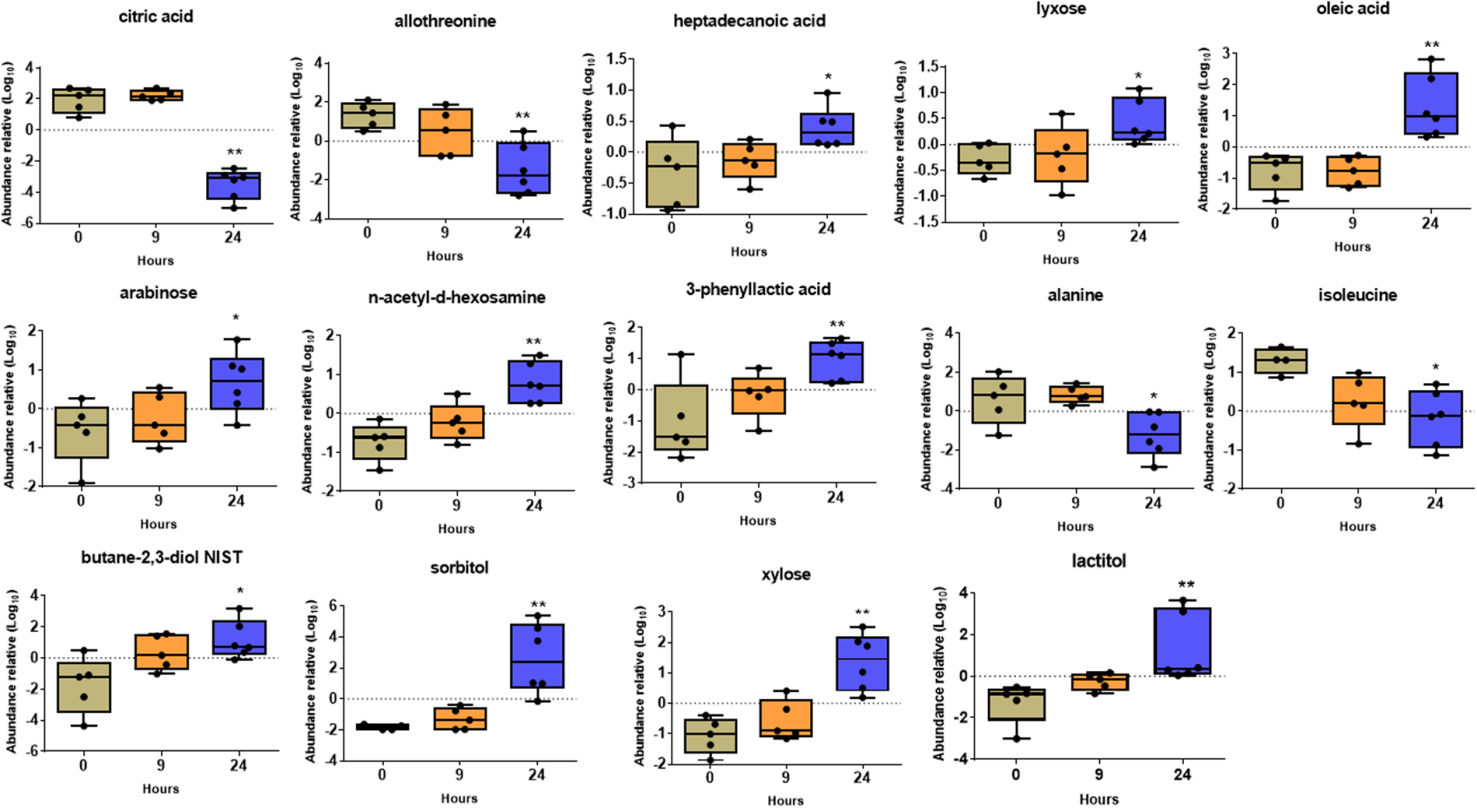
Changes in the relative abundance of metabolites in synovial fluid 24 h after the induction of acute ruminal acidosis (ARA). Box and whisker plot showing the median relative abundance. Each point represents an experimental animal. *p < 0.05; **p < 0.01 compared to 0 h; Dunn’s multiple comparison test.

After univariate analysis, we found 24 metabolites in synovial fluid that were significantly modified after oligofructose overload. At 9 h after the induction of ARA, significant increases in the levels of glyceric acid and cellobiose (∼30-fold), and fructose and lactic acid (17- and 16-fold, respectively) were observed. Moreover, the levels of erythritol, pyruvic acid, sucrose, maltose and galactitol were slightly increased (>1.5-fold) (Fig. 4). Threonine was the only compound to show reduced levels (4-fold) 9 h after oligofructose overload. At 24 h after the induction of ARA, significant increases were observed in the levels of sorbitol (49-fold), lactic acid and fructose (17- and 12-fold, respectively), lactitol, erythritol, butane 2,3-diol, oleic acid, glyceric acid, cellobiose, 3-phenyllactic acid, N-acetyl-D-hexosamine, arabinose, xylose, galactitol (>2-fold), and lyxose and heptadecanoic acid (>1.5-fold) (Fig. 5). In contrast, the levels of citric acid and threonine were significantly reduced 41- and 40-fold, respectively, whereas allothreonine and alanine were reduced >3.5-fold (Fig. 5).

### Metabolic pathway analysis

**Figure 6:**
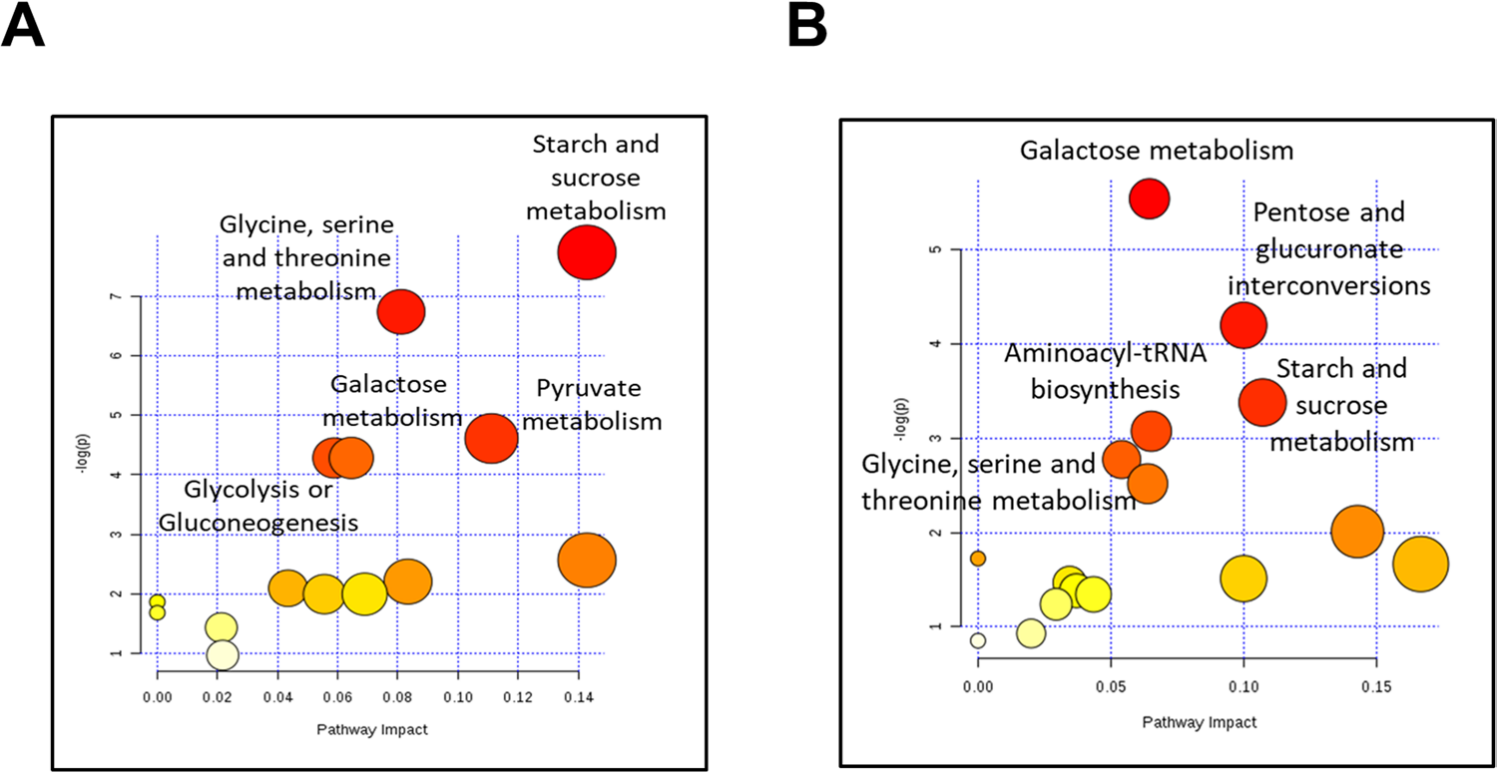
Metabolic pathway altered in synovial fluid after the induction of acute ruminal acidosis (ARA). All matched pathways according to the p-values from the pathway enrichment analysis and pathway impact values from the pathway topology analysis are shown. (**A**) 9 h and (**B**) 24 h after oligofructose overload.

We performed metabolic pathway analysis using Fisher’s exact test for over-representation of metabolites that were significantly modified at 9 h (Fig. 6A). At 9 h, the most significant pathways were “starch and sucrose metabolism”, “glycine, serine and threonine metabolism”, “pyruvate metabolism”, “galactose metabolism”, and “glycolysis or gluconeogenesis” (Fig. 6A). Pathway analysis of those metabolites that were significantly modified at 24 h (Fig. 6B) revealed changes in “galactose metabolism”, “pentose and glucuronate interconversions”, “starch and sucrose metabolism”, “-tRNA biosynthesis” and “glycine, serine and threonine metabolism” (Fig. 6B).

### Determination of lactic acid enantiomers by high-performance liquid chromatography (HPLC)

**Figure 7:**
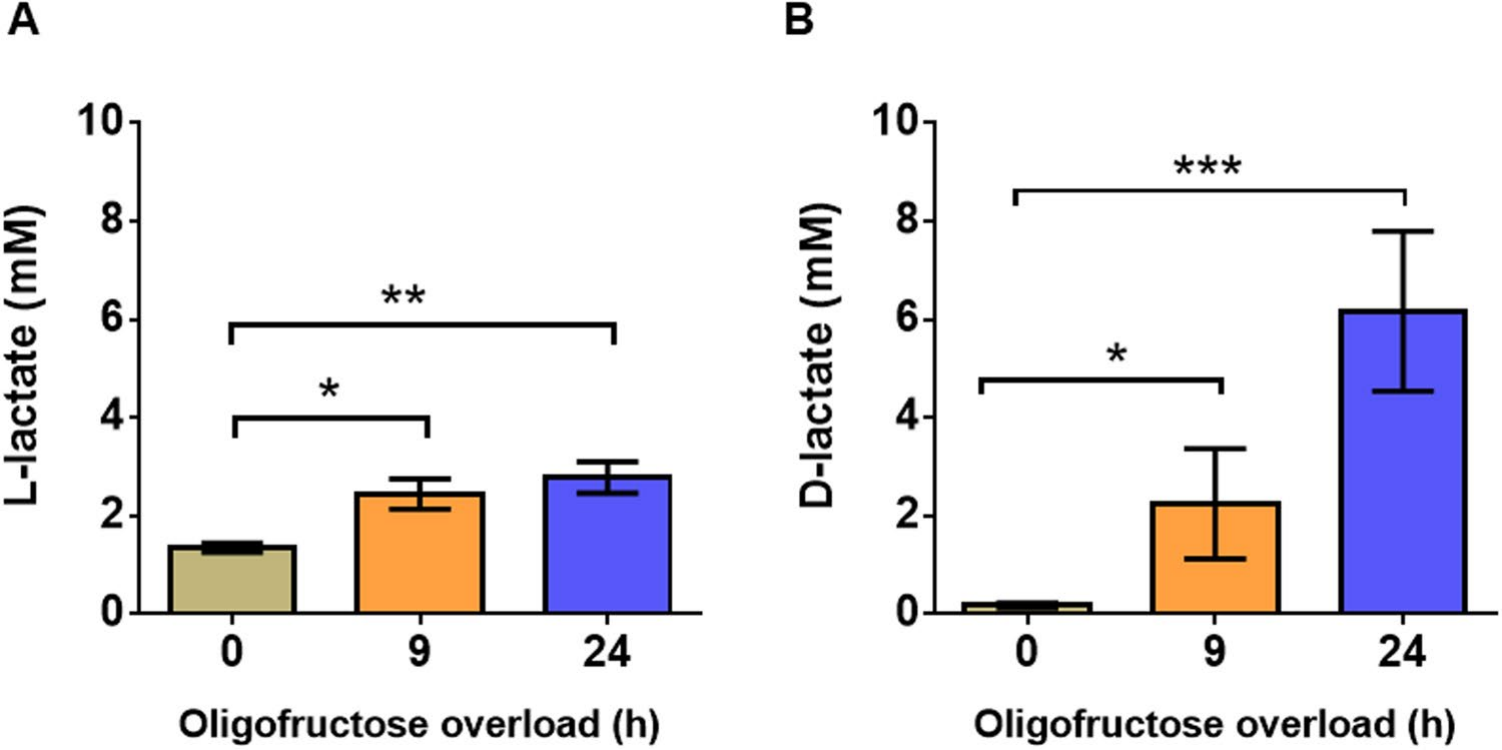
Presence of L- and D-lactate in synovial fluid from heifers with acute ruminal acidosis (ARA). (**A**) L-lactate and (**B**) D-lactate concentrations are shown. Each bar represents the mean ± S.E.M. *p < 0.05; **p < 0.01; ***p < 0.001 compared to 0 h; Dunn’s multiple comparison test.

At 0 h, the mean values of L- and D- lactic acid in synovial fluid were 1.4 and 0.2 mM, respectively. After oligofructose overload, L-lactic acid levels were significantly increased at 9 and 24 h, and the maximum mean value recorded in synovial fluid was 2.8 mM at 24 h (Fig. 7A). To an even greater extent, D-lactic acid levels were significantly increased at 9 and 24 h, with a comparatively higher mean value of 6.2 mM at 24 h after oligofructose overload (Fig. 7B).

### Increased interleukin-6 (IL-6) in the synovial fluid of heifers with ARA and FLS cells treated with D-lactic acid

**Figure 8:**
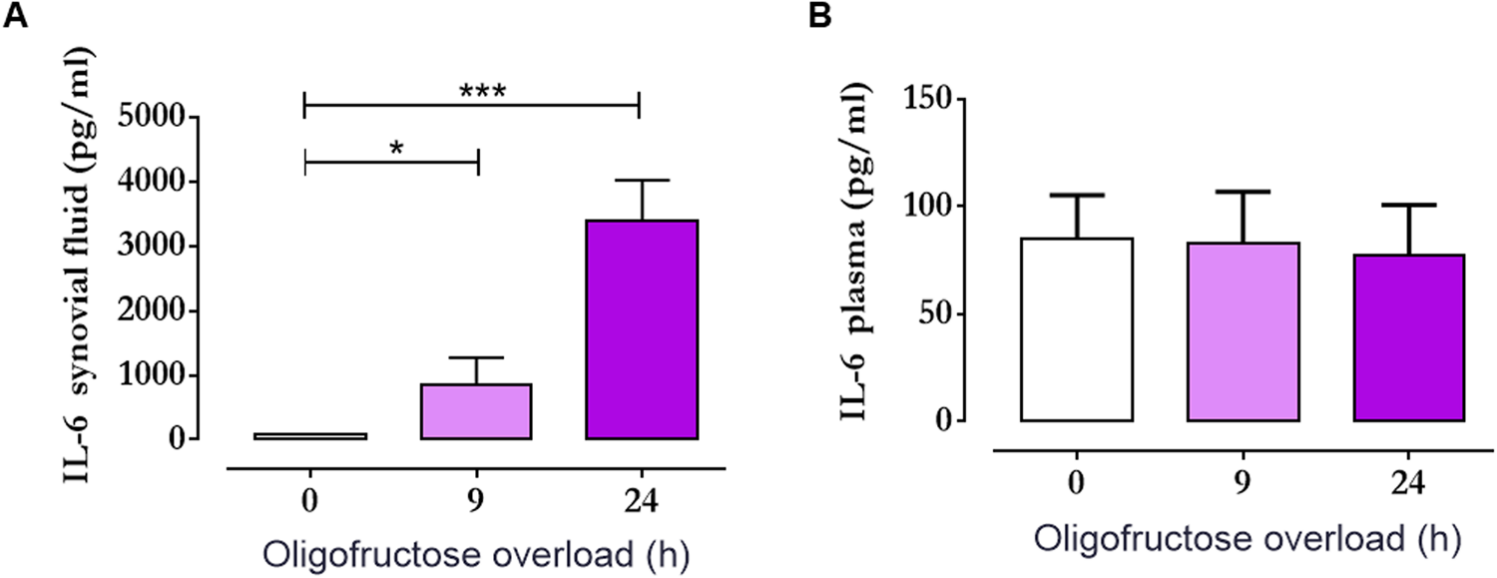
Interleukin-6 (IL-6) is increased in the synovial fluid, but not in the plasma of animals with acute ruminal acidosis (ARA). IL-6 concentrations in (**A**) the synovial fluid and (**B**) plasma of heifers challenged with oligofructose overload. Each bar represents the mean ± S.E.M. *p < 0.05; ***p < 0.001 compared to 0 h; Dunn’s multiple comparison test.

**Figure 9:**
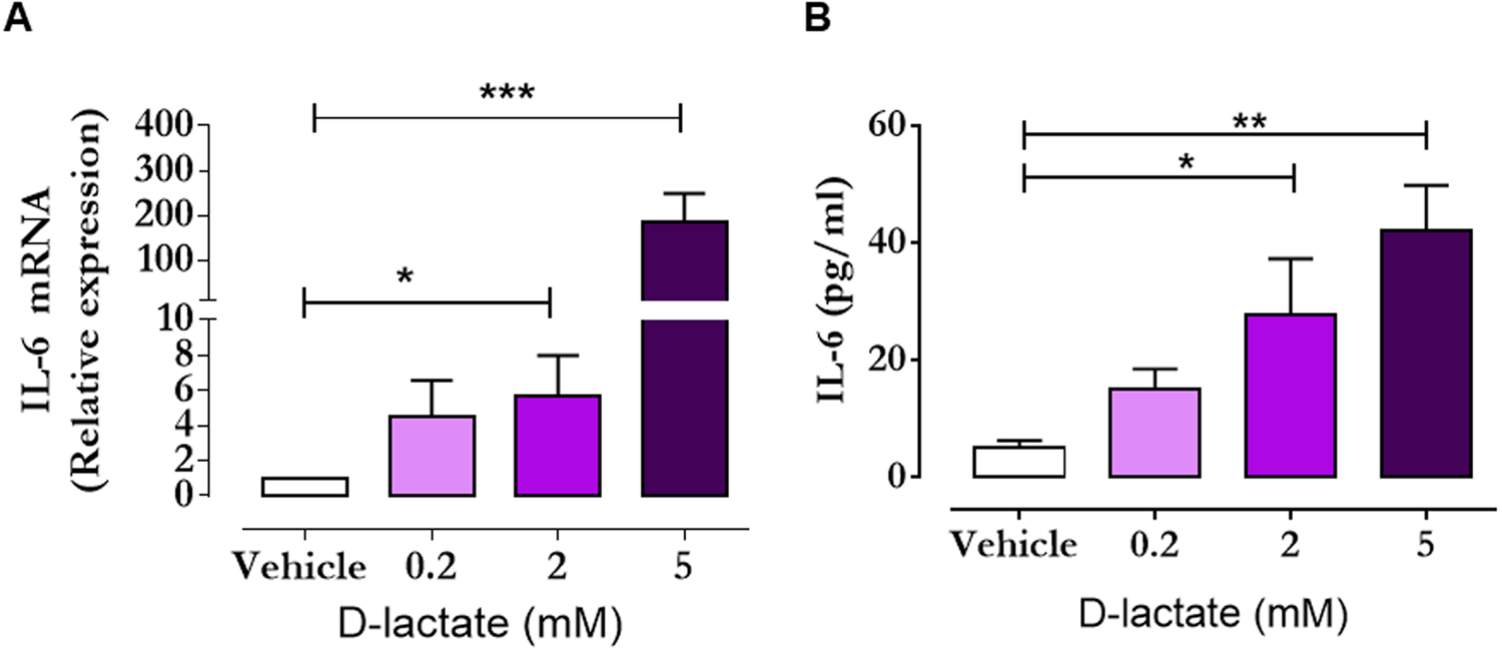
D-lactate stimulates the synthesis of interleukin-6 (IL-6) in bovine fibroblast-like synoviocytes (FLS). An increase in (**A**) the expression and (**B**) release of IL-6 induced by D-lactate are shown. Each bar represents the mean ± S.E.M. *p < 0.05; **p < 0.01; ***p < 0.001 compared to 0 h; Dunn’s multiple comparison test.

We observed a significant increase in the level of IL-6 in synovial fluid 24 h post-oligofructose overload (Fig. 8A). However, IL-6 in the plasma showed no change during induction of experimental ruminal acidosis (Fig. 8B), suggesting that IL-6 was only increased within the joint of the animal. We detected a significant increase in *IL-6* mRNA expression (Fig. 9A) and IL-6 release in the supernatant of FLS (Fig. 9B) treated with 2 and 5 mM D-lactic acid.

## Discussion

Ruminal acidosis is a common fermentative disorder in cattle, which induces comorbidities that include liver abscesses, parakeratosis, laminitis and polysynovitis^6,8^. In oligofructose overload experiments, an increase of tarsocrural joint inflammation is more evident clinically than claw sensitivity 24 h after acidosis induction^7^. Moreover, at 24 h post-oligofructose overload, the synovial fluid was turbid, with an abundance of leukocytes^8^. In the present study, we observed a similar pattern with a substantial increase in the number of polymorphonuclear leukocytes (PMNs) 24 h post-overload. Tarsal synovial fluid in cows is typically colourless and clear, with very few neutrophils^22^; therefore, this increase in the number of PMNs could be attributable to the acute inflammatory response observed in heifers with ARA^8^.

The ARA disorder is a well-known metabolic imbalance caused by excessive carbohydrate intake^6,23–25^. However, the metabolic changes in synovial fluid during synovitis induction by ARA have not yet been described. We used GC-MS for metabolite analysis, as the NIST14 library comprises mass spectra for 242,477 unique compounds. Among these compounds, roughly one third have recorded standardised retention times and are ideal for the identification and quantitation of small molecular metabolites (<650 Daltons), as compared to LC-MS/MS, the spectral libraries of which are significantly smaller in size and lack information on retention time^26^. The GC-MS approach revealed the presence of several chemical entities in synovial fluid before ARA, including glucose, mannitol, myristic acid, citric acid, urea, phosphate, stearic acid, 3-hydroxybutyric acid and glycine. In the synovial fluid of healthy humans analysed by GC/TOF MS, mannose, lactic acid, glucose, valine and citric acid were the most abundant chemical entities^27^.

The levels of several carbohydrates were increased in the synovial fluid of heifers treated with an overload of oligofructose. At 9 h after oligofructose overload, increased levels of cellobiose, maltose, sucrose and fructose were observed. Galactitol was increased at both 9 and 24 h after oligofructose overload. Furthermore, increased levels of sorbitol, arabinose, xylose and lyxose were observed in the joint 24 h after ARA induction. Thus, the key metabolic pathways within the joint, in which these metabolites were involved, were starch and sucrose, galactose metabolism, and pentose and glucuronate interconversions. Metabolomics profiling by UHPLC-QTOF/MS (ultra-high-performance liquid tandem chromatography quadrupole time of flight mass spectrometry) of the rumen contents and plasma of steers fed high-grain diets, revealed a high level of carbohydrates, such as D-maltose, D-mannose, D-Lyxose and sucrose^28^. In addition, starch and sucrose metabolism and galactose metabolic pathways were associated with metabolites that showed increased levels in the rumen^28^. Therefore, these findings suggest that heifers with ARA induced by oligofructose overload, or treated with high-grain diets, have similar metabolic alterations in the rumen, plasma and synovial fluid. In addition, the key metabolic pathways observed in the synovial fluid of heifers with ARA were correlated with those observed in the rumen of steers treated with high-grain diets^28^. The contribution of these metabolic pathways to inflammatory processes of the joint have been poorly understood. Nevertheless, galactitol and sorbitol have been associated with joint damage through potential osmotic stress in diabetic patients^29^, but their importance in the pathogenesis of synovitis is unknown.

The levels of several amino acids were slightly reduced in the synovial fluid of animals with ARA. The reduced levels of threonine and allothreonine could indicate an increased rate of nucleotide synthesis^30^; whereas thee inhibition of alanine and isoleucine, two branched-chain amino acids, could be indicative of regulation of the inflammatory process^31^. However, the reasons for and relevance of these observations are challenging to deduce. We observed at different times that all routes converged to elevate the levels of lactic acid in the joints of heifers with ruminal acidosis.

At 9 h post-oligofructose overload, the increase in glyceric acid, pyruvic acid and lactate was suggestive of an up-regulation in the pyruvate metabolic pathway in the joint. Metabolomic analysis by GC/TOF MS of the synovial fluid of patients with RA has also revealed an increase in the levels of fructose^32^, glyceric acid^30,32^ and lactate^27,32^. Increased lactate in the synovial fluid of human patients with arthritis has been previously described^9,13,27^. Similar metabolic changes have also been observed in other anatomical compartments. Metabolomic analysis by GC-MS of ruminal fluid from cows with acidosis revealed an increase in the levels of pyruvate and lactate^33^. A more substantial increase in lactate and reduction in citrate were observed within the joint 24 h after the induction of ARA, suggesting that the tricarboxylic acid (TCA) pathway and aerobic oxidation were attenuated in the joints of animals with ARA. Similar findings have been described in the synovial fluid of patients with RA, as analysed by GC/TOF MS, which showed a down-regulation of enzymes associated with the TCA pathway^27,34^.

Similar to previous reports, we observed no microorganisms in the synovial fluid, suggesting that animals with ARA induced by oligofructose overload developed aseptic synovitis^8^. Thus, the increases in lactate and pyruvate could have been related to increased glycolysis in the inflamed joint^35^. However, we detected no changes in glucose and pyruvate at 24 h post-oligofructose overload, suggesting that other metabolic sources could have been involved.

There is evidence of crosstalk between inflammatory processes and metabolic dysregulation^36^. During the inflammatory process, cells undergo metabolic changes, such as the shift toward increased glucose uptake and glycolysis^37^. Other authors have proposed that the pyruvate dehydrogenase kinase (PDK)-pyruvate dehydrogenase (PDH)-lactic acid axis is involved in inflammatory processes^38^. Previous research suggests that within the site of inflammation, there is an alteration in the PDK-PDH lactic acid axis, thereby decreasing the oxidation of pyruvate and increasing its conversion to lactate^38^. In addition, this metabolic change associated with lactic acid production, induces the recruitment of inflammatory cells to the site of the inflammation^38^. However, the contribution of this metabolic alteration to the onset of synovitis during ARA is yet to be fully understood.

During ARA, the levels of both D- and L-lactate are increased in the ruminal fluid and plasma of cattle^5,39^. Previously, we demonstrated an increase in lactate in the plasma of heifers 8 h after oligofructose overload^19^. D-Lactate is normally present in the blood of mammals at nanomolar concentrations owing to methylglyoxal metabolism^40,41^; millimolar D-lactate concentrations can arise because of an excess in gastrointestinal microbial production. Grain overload in ruminants, the equivalent of short bowel syndrome in humans, and diarrhoea in calves can lead to profound D-lactic acidaemia^42^. As the metabolomic analysis showed no differences between lactic acid enantiomers, we performed HPLC analysis of the synovial fluid using a cationic exchange column that separated lactic acid enantiomers. At 9 and 24 h post-oligofructose overload, we observed that the levels of both lactate enantiomers were increased. Furthermore, D-lactate (⁪6 mM) showed the highest concentration in the synovial fluid of heifers with ARA. The increase in D-lactate could be attributed to the presence of ruminal bacteria, such as *Streptococcus bovis* and *Lactobacillus sp*., and was the only enantiomer that is significantly increased in blood during ARA^5,6^. In addition, 24 h after oligofructose overload, D-lactate levels in synovial fluid were similar to those detected in the plasma of bovines with ARA^5^. In cattle with ARA, D- and L-lactate can both be distributed in other anatomical compartments *e.g*. cerebrospinal^43^. The presence of relatively low and similar concentrations of D-lactate (>0.15 mM) has been observed in the synovial fluid of humans with aseptic arthritis or bacterial synovitis; nevertheless, the diagnostic significance of this finding is debatable^9,44^. In contrast, higher concentrations of D-lactate in the joint could be more closely associated with the diagnosis of synovitis in animals with ARA.

Joint inflammatory processes also contribute to increased lactate and metabolic disturbances^45,46^. Thus, a more specific experimental design is required to demonstrate that alterations in the levels of lactic acid (or other metabolites) in the joint are exclusively derived from ruminal disorders and produced by microbiota^6^. In *in vitro* experiments, D-lactate increases neutrophil extracellular traps, PMN endothelial adhesion and MMP-9 release^47,48^, suggesting that D-lactate could exert pro-inflammatory effects in the joint. Bovine polysynovitis induced by oligofructose overload is an aseptic inflammatory process^8^. As IL-6 is a pro-inflammatory cytokine that is increased in aseptic joint diseases^49–51^, we assessed the levels of IL-6 in the synovial fluid and plasma of heifers with ARA. We detected significantly increased levels of IL-6 in the synovial fluid at 9 and 24 h post-oligofructose overload, but not in the plasma, suggesting that the inflammatory process in the joint was an early event closely associated with the presence of D-lactate. In RA, the presence of relatively high concentrations of lactate is an established hallmark of the pathogenesis of the disease^13,27,52^. Moreover, the cellular metabolic disturbances and mitochondrial dysfunction observed in the joint actively induce inflammation and contribute to the proliferation of the synovial membrane and articular cartilage, and bone damage^45,46^. Metabolic profiling of the FLS of patients with RA has demonstrated increases in glucose metabolism (glycolysis and the pentose phosphate pathway) and amino acid metabolism (tyrosine and catecholamine biosynthesis and protein biosynthesis)^53^, thus contributing to the increased production of lactate^27,54^. In the synovium, FLS comprise the key cellular component associated with the onset of inflammatory process that produces pro-inflammatory cytokines such as IL-6^55,56^. Lactate can exert a role as a second messenger in the synthesis of cytokines during RA^15^. In view of the fact that D-lactic acid was the main enantiomer to be increased within the joints of animals with experimental ruminal acidosis, and was correlated with an increase in IL-6 in the joints of heifers with ARA, we assessed whether D-lactic acid was able to induce IL-6 production *in vitro* using bovine FLS. We demonstrated that D-lactate increased the expression and release of IL-6 in a dose-dependent manner, suggesting that the presence of this metabolite in the joint contributes to the onset of synovitis in cattle.

We conclude that heifers with ARA induced by an oligofructose overload present metabolic changes in the synovial fluid, characterised by an increase in lactate, particularly of the D-enantiomer, prior to the appearance of synovitis. Further research that includes the analysis of synovial fluid from animals subjected to various types of livestock systems, and those affected by distinct levels of ruminal acidosis, would contribute to the establishment of the role of metabolomic disturbances in the pathogenesis of inflammatory joints and lameness in cattle.

## Methods

### Animals and housing

Seven nonpregnant black Friesian dairy heifers, aged between 10 and 18 months, and weighing between 200 and 250 kg, were selected. The animals were obtained from the Estación Experimental Agropecuaria Austral farm of the Austral University of Chile, and were housed in the large animal facilities of the Veterinary Hospital of the Austral University of Chile. The health status of the animals was verified through a clinical examination and complementary tests (haemogram and biochemical parameters). No remarkable deviations were observed in rectal temperature, heart and respiratory rate, frequency of ruminal contractions, haematocrit, red blood cell count, other blood cell counts, or glucose, lactate dehydrogenase (LDH) and albumin in the plasma, that suggested any alterations in the health status of the animals. In addition, the animals were free of brucellosis, leucosis and tuberculosis, and were certified by the National Livestock Service of Chile. The animals were acclimated for a 4-week period before initiating the experiments and carefully handled to avoid stress throughout the experiments.

The heifers were fed twice daily. The daily ration of concentrate was equally divided into two meals of 1.0 kg/d each of Cosetan® (IANSAGRO S.A., Chile), and water was provided *ad libitum*. The heifers grazed on naturalised pasture composed primarily of perennial grasses, mostly *Holcus lanatus* and *Agrostis capillaris*. The contribution of forage legumes was low, at <10% of the dry matter.

### Oligofructose overload

Oligofructose (13 g/kg BW) (Beneo P95, Orafti Active Food Ingredients, Santiago, Chile) dissolved in warm tap water was administered in a volume of 2 L/100 kg of BW as a ruminal drench, as previously described^19,57^. All animals were clinically monitored (heart and respiratory rate, rectal temperature, ruminal frequency and signs of laminitis) by a veterinary clinician, and all procedures were performed in the ruminant unit of the Veterinary Hospital of Universidad Austral de Chile. An increased heart rate was observed at 9 and 24 h, whereas the frequency of ruminal contractions was reduced (<1/min) 24 h after oligofructose overload. In addition, at 9 and 24 h, all animals treated with oligofructose overload showed lameness, resulting in a locomotion score of 2–3^58^.

Twenty-four hours after the administration of the total dose of oligofructose, a solution of sodium bicarbonate (1 g/kg BW) and ruminal restorative mineral salts (Bilifar®, DragPharma) was administered orally, and a solution of an analgesic/anti-inflammatory (Febrectal®, DragPharma) and antibiotic (Pencidrag ®, DragPharma) was administered by the intramuscular route. At the end of the study, all animals recovered, and none exhibited secondary pathologies after 2 months of observation.

All experiments were conducted in accordance with the Institutional Review Board-approved protocols, the National Guidelines on the Use of Experimental Animals of the Comisión Nacional de Ciencia y Tecnología (CONICYT) de Chile, and the Guidelines for the Use of Animals in Research of the Directorate of Research and Development of the Universidad Austral de Chile (N° Bioethics Report 217/2015 and N° bioethics monitoring S-49-2017).

### Collection of ruminal and synovial fluid samples

During the 24 h following the administration of oligofructose, samples of ruminal fluid and synovial fluid were collected from the animals. Three schedules were followed for the collection of samples: H 0, which corresponded to the sample collected before the administration of the total dose of oligofructose; H 9, which corresponded to the sample collected 9 h after the administration of the total dose of oligofructose; and H 24, which corresponded to the sample collected 24 h after the administration of the total dose of oligofructose. Rumenocentesis was performed in the dorsal sac of the rumen to collect ruminal fluid^59^. The pH of the ruminal fluid was quickly measured to assess the experimental procedures and confirm the presence of acidosis.

To obtain the synovial fluid, aseptic arthrocentesis was performed as previously described^60,61^. The incisions were made only once in each joint to avoid contamination of the samples. The incision sites were located in the *talus* and *os centroquartale* of the tarsal joint, and between the intermediate and lateral patellar ligaments of the knee joint. The samples were analysed separately.

We excluded samples that showed the presence of blood, as obtained during the incision into the joint, to minimise any analytical bias in the detection of metabolites in synovial fluid and in IL-6 ELISA detection. In addition, only one sample per day was collected from each joint, as approved by the institutional bioethical committee.

The pH of the synovial fluid was measured immediately after sample collection by a portable pH metre (Hanna Instruments, USA). In addition, cellular smears were stained with Giemsa, and differential counts were performed. Synovial fluid was centrifuged at 1000 × *g* for 10 min at room temperature, and the supernatant was collected and stored at −80 °C until the performance assay.

### Quantification of L- and D-lactate in synovial fluid by HPLC

Synovial fluid was loaded into Amicon® Ultra-4 3 K (Merck Millipore, Darmstadt, Germany) tubes and centrifuged at 75 000 × *g* for 40 min at 4 °C. The filtrates were concentrated in a SpeedVac concentrator (Savant® SPD131DDA, Thermo Fisher Scientific, USA) for 90 min at 45 °C and 1.5 atmospheres of pressure. The concentrates were suspended in 200 µL of 1 mM CuSO_4_ (mobile phase) and analysed using HPLC, as previously described^48^.

### Sample preparation for metabolomics analysis

An aliquot of 100 µL of synovial fluid was extracted with 1 mL of cold HPLC-grade acetonitrile/HPLC-grade isopropanol/HPLC-grade water (3:3:2 vol/vol/vol) containing 1 µL of ribitol (Sigma-Aldrich, Chile) in water (0.4 mg/mL) as an internal standard. The samples were vortexed for 10 s and kept at 4 °C for 5 min in a shaker, and then centrifuged at 14 000 × *g* for 2 min at room temperature. After centrifugation, 450 µL of the supernatant was evaporated to dryness using a SpeedVac concentrator (Savant® SPD131DDA, Thermo Fisher Scientific, USA). Subsequently, 450 µL HPLC-grade acetonitrile/HPLC-grade water (1:1 vol/vol) was added and vortexed for 10 s, centrifuged at 14 000 × *g* for 2 min at room temperature, and the supernatant was evaporated to dryness using a SpeedVac concentrator. The FAME mix (2 µL) (C8–C30) (400505, Fiehn GC/MS metabolomics standards kit, Agilent, CA, USA) was used as retention index markers. In addition, 10 µL methoxyamine hydrochloride (MeOX)/pyridine (20 mg/mL, Sigma-Aldrich, Chile, in American Chemical Society (ACS)-grade pyridine) were added to dry samples and kept at 30 °C for 90 min in a shaker. Subsequently, 90 µL MSTFA (*N*-methyl-*N*-trifluoroacetamide) with 1% TMCS (trimethylchlorosilane) derivatisation agent (Thermo Fisher Scientific, Pierce Biotechnology, Rockford, IL) was added, and the mixture was incubated at 37 °C for 30 min on a hot plate and vortexed three times during incubation to ensure complete dissolution. The samples were transferred to a glass vial insert (250-µL, Agilent, Santa Clara, CA) in a 1.5-mL glass vial with screw cap (Agilent) for GC-MS analysis.

### Metabolomics analysis by GC-MS

Derivatised samples were injected with an Agilent 7693 Series Autosampler (Agilent Technologies, Palo Alto, CA), after which they were analysed by an Agilent 7890B GC system coupled to an electron impact (EI) ionisation mode 5977 A mass selective detector (Agilent Technologies). A 1-µL aliquot of each derivatised sample was injected in a splitless injector mode onto a 30 m × 0.25 mm × 0.25 µm DB-5 column (Agilent Technologies). The injector port temperature was held at 250 °C, and the helium carrier gas flow rate was set to 1 mL/min at an initial oven temperature of 60 °C. The oven temperature was increased at 10 °C/min to 325 °C for a final run time of 37.5 min. Full-spectra/s (50–600 m/z; 1.7 scans/s) with a digital scan rate of 20 Hz were acquired after a 5.9-min solvent delay, with an MS ion source temperature of 250 °C and quadrupole temperature of 150 °C.

All derivatised samples were run within 24 h after preparation. A mixture of the FAME standard solution C8–C30 (Agilent, USA) was injected to obtain the retention times for calculation of the Fiehn’s retention index of metabolites.

Raw MS data (‘.D’ file format) were first transformed into the Analysis Base File (ABF) format by the software *Reifycs* ABF Converter prior to data pre-treatment. Identification of metabolites was performed, following methods previously described^26^. Briefly, peak detection, deconvolution and peak alignment in data processing were performed using the MSDIAL 2.83 software to process the total ion chromatogram and the EI–MS spectra of each GC peak. After deconvolution, the purified mass spectrum of each of the trimethylsilylated metabolites was identified, and deconvoluted peaks were matched against mass spectral libraries that were imported in the National Institute of Standards and Technology (NIST) MSP format. Library match hits were ranked against experimental data based on the total retention index and mass spectral similarity across all samples that were processed in a batch. The retention index, Fiehn RI, based on FAME, was used. Metabolites were identified by matching the EI–MS spectra with those of reference compounds from the NIST or Fiehn libraries. For the analysis, we used a 2000 retention index tolerance, 70% EI similarity cut-off, 70% identification score cut-off, 0.5 Da m/z tolerance and 0.5 min retention time tolerance.

### Cell culture of synoviocytes

Bovine fibroblast-like synoviocytes (bFLS) (# CDD-B-2910, Articular Engineering, USA) were cultured in a sterile 25-cm^2^ plastic tissue culture flask (# 70025, SPL, Korea) with synoviocyte growth medium (# M-2700, Articular Engineering, USA) at 37 °C and 5% CO_2_. In the fourth passage, the bFLS were cultured in sterile 6-well plates (# 31006, SPL, Korea) in Dulbecco’s modified Eagle’s medium (DMEM) F12 (#12400016, Gibco, USA) with 10% FBS (# 10437028, Thermo Fisher Scientific, USA) and stimulated with different concentrations (0.2, 2 and 5 mM) of D-lactic acid (# L0625, Sigma-Aldrich, Chile) for 6 h. Four replicates were used in the analysis.

### Real-time PCR for *IL-6* mRNA

Total RNA from FLS treated with D-lactic acid was extracted with a EZNA kit (E.Z.N.A., Promega, USA). The samples were treated using the Turbo DNase-Free® kit (Thermo Fisher Scientific, USA). For cDNA synthesis, 700 ng of total RNA was reverse transcribed using a QPCR Affinity Script® cDNA synthesis kit (Agilent Technologies, USA.). Real-time PCR assays were performed using a reagent mixture of Master qPCR SYBR Green® (Fermentas Life Sciences, USA). The forward primer for IL-6 was 5′ACTGGCAGAAAATAAGCTGAATCTTC3′, and the reverse primer was 5′TGATCAAGCAAATCGCCTGAT3′. The *IL-6* mRNA levels were normalised with succinate dehydrogenase complex flavoprotein subunit A (SDHA) using the forward primer 5′GCAGAACCTGATGCTTTGTG3′ and reverse primer 5′CGTAGGAGAGCGTGTGCTT3′.

The following conditions were used in a qPCR StepOne™ system (Thermo Fisher, USA): 35 cycles at 95 °C for 30 s, 60 °C for 60 s (annealing), and 72 °C for 60 s (extension). The expression changes were calculated using the 2^−(ΔΔCt)^ method, according to Livak and Smittgen^62^, using the StepOne™ v2.3 software.

### IL-6 ELISA

To measure IL-6 in the synovial fluid of heifers with experimentally induced ruminal acidosis, the synovial fluid was centrifuged at 1000 × *g* for 10 min, and 200 µL of the supernatant was collected. In addition, 200 µL of the supernatant of the bFLS culture was also analysed. In addition, 8 mL of blood was collected by jugular venipuncture into acid citrate dextrose (ACD)-lined collection tubes (Becton Dickinson, USA) and then centrifuged at 1000 × *g* at 20 °C for 20 min to obtain 200 µL of plasma for further analysis. The bovine IL-6 ELISA kit (# ESS0029, Thermo Fisher Scientific, USA) was used to estimate IL-6 concentrations according to the manufacturer’s instructions. The samples were analysed at 450 nm and 550 nm in an automated Varioskan Flash reader (Thermo Fisher Scientific, USA).

### Statistical analysis

Univariate analysis of continuous data was performed using the Kruskal–Wallis ANOVA and Dunn’s multiple comparison test and Prism v5.0 OS-MAC software. All multivariate metabolomics analyses were processed statistically using the MetaboAnalyst software (http://www.metaboanalyst.ca/)^63^. Recommended statistical procedures for metabolomics analysis were followed, according to previously published protocols^63^. Metabolites that were frequently (>50%) below the limit of detection, or with at least 50% missing values were removed from consideration. Data normalisation of metabolite concentration was performed using ribitol as an internal standard, and logarithmic transformation and auto scaling were performed prior to statistical analysis to create a Gaussian distribution^63^. The PCA, PLS-DA and VIP scores were determined. The PLS-DA model was validated by cross validation and permutation tests, as the sum of squares captured by the model (R2) > 0.9 and the value of *P* = 0.0285 (57/2000), respectively.

Heat maps were depicted, and to retain the most contrasting patterns, a Euclidean distance measure and Ward’s clustering algorithm were used. Metabolites were then studied to generate integrated pathway enrichment and pathway topology analyses. *Bos taurus* (cow) was selected as the model organism.

Pathway enrichment and pathway topology analyses were performed using the *Bos taurus* pathway library and a hypergeometric test for over-representation analysis. For the identification of potential metabolomic pathways, biochemical databases, including the Human Metabolome Database (HMDB) (http://www.hmdb.ca/), Kyoto Encyclopedia of Genes and Genomes (KEGG) (http://www.genome.jp/kegg/) and the Bovine Metabolome Database (http://www.cowmetdb.ca/), were used.

## Supplementary information

Supplementary table and figure legends

Supplementary table S1
