# Supplementary material for: Metabolic disturbances in synovial fluid are involved in the onset of synovitis in heifers with acute ruminal acidosis
Source: Sci Rep. 2019 Apr 1;9:5452. doi: 10.1038/s41598-019-42007-1 (PMC6443794; doi:10.1038/s41598-019-42007-1)

Metabolic disturbances in synovial fluid are involved in the onset of synovitis in heifers with acute ruminal acidosis.

Pablo Alarcon[1], Alejandra A Hidalgo[1], Carolina Manosalva[2], Raul Cristi[1], Stefanie Teuber[1], Maria A Hidalgo[1], Rafael A Burgos[1] *.

[1]Laboratory of Inflammation Pharmacology, Faculty of Veterinary Science, Institute of Pharmacology and Morphophysiology, Universidad Austral de Chile, Valdivia, Chile

[2]Faculty of Sciences, Institute of Pharmacy, Universidad Austral de Chile, Valdivia, Chile

*Correspondence to:
Rafael A Burgos: rburgos1@uach.cl

Supplementary table S1. Retention time, retention index, quant mass, metabolite name, and total similarity of the compounds detected by GC-MS of synovial fluid before ARA induction.

Supplementary fig S1. Partial least square-discriminant analysis (A) and the greatest variable importance in projection scores (B) of metabolites of synovial fluid from heifers with ARA are shown.

Supplementary fig S1

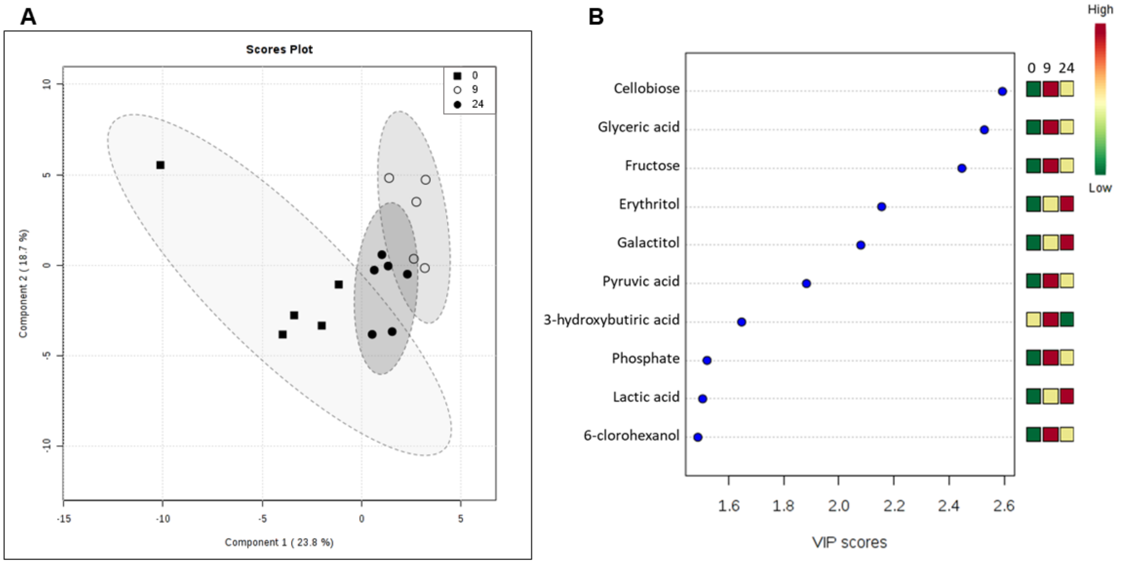

Supplement: Supplementary file 1 — Supplementary table and figure legends [file 41598_2019_42007_MOESM1_ESM.pdf]
